# Study on the Chloride–Sulfate Resistance of a Metakaolin-Based Geopolymer Mortar

**DOI:** 10.3390/ma17205045

**Published:** 2024-10-15

**Authors:** Jiangbo Cheng, Yongjun Qin, Ziqi Yao, Ling Luo, Changwei Qu

**Affiliations:** College of Civil Engineering and Architecture, Xinjiang University, Urumqi 830047, China; 107552204529@stu.xju.edu.cn (J.C.); 107552104185@stu.xju.edu.cn (Z.Y.); luoling@xju.edu.cn (L.L.); changweiup@stu.xju.edu.cn (C.Q.)

**Keywords:** geopolymer mortar, immersion method, chloride–sulfate erosion, mechanism of durability

## Abstract

The chloride–sulfate corrosion environment of concrete is a significant engineering problem. This paper investigates the effect of the complete/semi–immersion mode on the durability of concrete in a chloride–sulfate environment by using different granulated blast furnace slag (GBFS) dosage rates (10–50%) of a metakaolin (MK)-based geopolymer mortar. The chloride–sulfate corrosion environment is discussed by analyzing the apparent morphology, mass change, and mechanical property change in specimens at the age of 120 d of erosion combined with XRD and SEM. The high Ca content in GBFS has an important effect on the strength and erosion resistance of the metakaolin geopolymer (MGP) group mortar; an increase in the GBFS dosage makes the MGP group mortar denser, and the initial strength of the MGP group mortar is positively correlated with the dosage of GBFS. After 120 d of erosion, the GBFS dosage is negatively correlated with erosion resistance, with the high GBFS dosage groups showing more severe damage. Semi-immersion resulted in more severe deterioration at the immersion–evaporation interface zone due to the difference in the ionic concentration and the ‘wick effect’ at the immersion–evaporation interface zone. Compared with the commonly used OPC mortar, the M40 and M50 groups have improved strength and corrosion resistance and are suitable for engineering environments in highly erosive areas.

## 1. Introduction

Ordinary Portland Cement (OPC) is considered one of the most important construction materials and has good mechanical properties with low cost. However, the manufacturing of OPC consumes a large amount of non-renewable resources and releases greenhouse gases, which cause irreversible damage to the natural environment [1,2,3]. Therefore, there is an urgent need to replace the manufacturing of OPC with lower energy consumption and environment-friendly materials to realize sustainable development.

Geopolymers, produced from industrial by-products or calcined clay, are gaining interest as environment-friendly cementitious materials to replace OPC and have received much attention in recent years. Furthermore, geopolymers exhibit many excellent properties, such as high early strength, high-temperature resistance, and good durability [4,5,6,7]. Metakaolin (MK) is considered the material of choice for geopolymers because of its unordered arrangement of molecules and richness in Al and Si [8,9]. However, in other studies, MK is obtained by calcined kaolin at a high temperature, which has low activity at low temperatures and is economically expensive to use [3,10,11]. Studies have shown that granulated blast furnace slag (GBFS), as an industrial by-product, can stimulate the activity of Al and Si in MK and form ${{\mathrm{SiO}}_{4}–\mathrm{Ca}–\mathrm{SiO}}_{4}$ bonds. Subsequently, dense C–(A)–S–H- and N–A–S–H-type reaction products are generated, which results in an MK–based geopolymer with low porosity and high strength [12].

Saline soils and acid rain lakes in many regions, such as the northwest region of China, can affect mechanical properties and durability significantly; this chloride–sulfate erosion greatly affects engineering constructions with potential safety hazards [13,14,15,16]. Chloride and sulfate erosion are two of the most prevalent durability problems in northwest China. At present, the effect of chloride and sulfate erosion on the mechanical properties of concrete has been investigated in many studies. Chloride is virtually non-erosive to concrete [17,18,19]. However, the effect of chloride on the corrosion of steel reinforcementcan cause the irreversible performance degradation of reinforced concrete structures [20,21,22]. Chloride is generally present in the following main forms: one is ${\mathrm{Cl}}^{-}$ bound to calcium sulfoaluminate hydrates, producing Friedel’s salt, while the other is mainly ${\mathrm{Cl}}^{-}$ adsorbed on C–(A)–S–H gel and exists in the pores of concrete [23,24]. Sulfate damage to concrete is mainly a chemical reaction that forms gypsum, ettringite (AFt), and other crystalline expansion damage [25,26]. When chloride and sulfate coexist in concrete, the erosive environment is more complicated. Jin and Jian [27,28] found that the presence of sulfate in the pre-erosion stage hindered ${\mathrm{Cl}}^{-}$ diffusion, but in the later stage of the composite solution, the presence of sulfate led to an increase in chloride, and Bo and others [29,30,31] found that chloride inhibits the formation of AFt, which slows down the expansion of sulfate damage. It has also been suggested that ${\mathrm{Cl}}^{-}$ promotes concrete expansion and cracking, thus accelerating the corrosion of concrete by ${\mathrm{SO}}_{4}^{2-}$ [32,33,34]. Although many studies have focused on the physical and chemical interaction between chloride and sulfate, the effect of chlorine and sulfate coexistence on the corrosion mechanism in MK-based geopolymer needs to be deeply investigated. However, different research studies still have different conclusions on the erosion mechanism in the environment of coexisting chloride–sulfate erosion [27,35,36,37,38,39]. Further, because of changes in the water table, building structures are often under complete/semi-immersion. It is important to study the effect of different types of immersion methods on chloride–sulfate composite erosion in MK-based geopolymer materials. The semi-immersed environment may lead to different erosion effects in the immersion–evaporation interface zone due to changes in ions transport [40,41,42].

This study is based on the existence of a large amount of ${\mathrm{Cl}}^{-}$ and ${\mathrm{SO}}_{4}^{2-}$ in the saline soil environment. The structures and properties after 120 d of erosion in 5%${\mathrm{Na}}_{2}{\mathrm{SO}}_{4}$ + 5%NaCl solution of MK-based sodium silicate with a 10–50% GBFS admixture under other fixed parameters were studied. This study aimed to explore the deterioration mechanism of MK-based geopolymer mortar with different GBFS dosages and different immersion methods. A possible hypothesis is that the reduction in GBFS content results in a lower calcium content, resulting in a looser structure of the MGP, whereas during the erosion process, erosion products (e.g., AFt) fill the pores of the matrix, resulting in a more dense structure, which may cause slight swelling damage in the later stages of erosion, resulting in an insignificant decrease in the strength of the low-GBFS dosage MGP group. The above-mentioned tests and hypothesis would provide technical support for the application of geopolymers in highly erosive areas.

## 2. Materials and Methods

### 2.1. Materials

**Figure 1:**
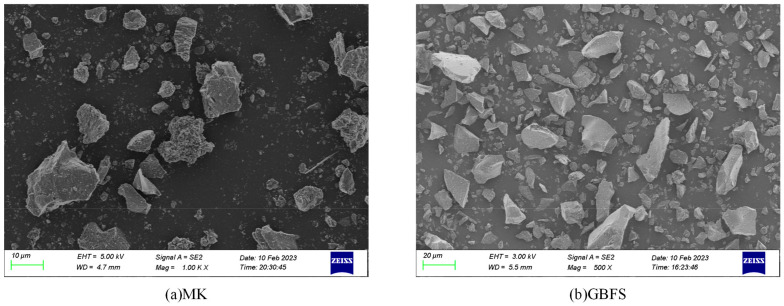
SEM images of MK and GBFS used as raw materials.

The raw materials used in the experiment are as follows: P–O42.5R cement purchased from Xinjiang Urumqi Tianshan Cement Plant was used as OPC. Geopolymer precursors were selected from MK and ground GBFS, and their SEM images are shown in Figure 1. MK was selected from 1250 mesh low-temperature calcined (750 °C~850 °C) MK produced by Hebei Shifeng Mining Co., Ltd. (Lingshou, China), and GBFS was produced by Hebei Shifeng Mining Co., Ltd. (Lingshou, China), with an activity index of 101%, a specific surface area of about 430, and an alkalinity coefficient of $K_{b}$ = 1.23, which is alkaline slag. The chemical composition of the MK and GBFS used as raw materials is presented in Table 1.

### 2.2. Mix Proportions

**Figure 2:**
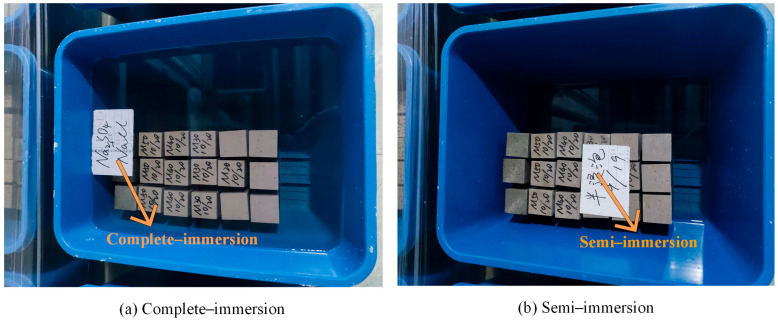
Complete/semi-immersion methods.

The geopolymer mortars were 40 mm × 40 mm × 160 mm prismatic specimens, mixed according to the GB/T 17671–2021 [43]. After 28 d curing, they were eroded under complete/semi-immersion in chloride–sulfate erosion for 120 d. Details of the complete/semi-immersion methods are shown in Figure 2.

**Figure 3:**
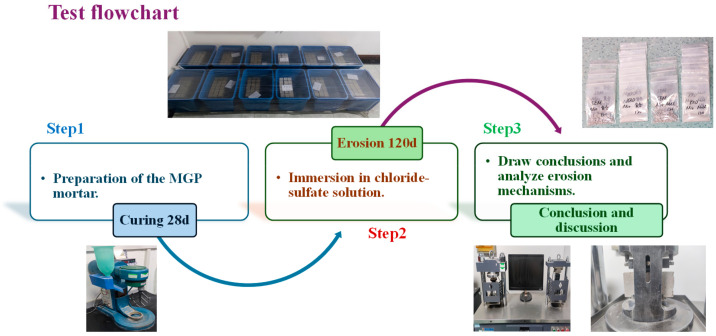
Test flowchart.

The mix proportions are shown in Table 2. The mixes with geopolymer are referred to as MGP groups. The alkali activators used in the test were prepared from sodium silicate solution and sodium hydroxide at room temperature. The sodium silicate solution was purchased from Urumqi Anning Drainage Jinxin Sodium Silicate Factory, and the sodium hydroxide reagent was analytically pure and purchased from Tianjin Xinbute Chemical Co. The use of industrial-grade sodium silicate modulus is ineffective, resulting in increased porosity and shrinkage. Meanwhile, the ability of the matrix to resist deformation decreased. The strength of the mortar is enhanced by stimulating the activity of Al and Si in the geopolymer. Therefore, the industrial-grade sodium silicate needs to reduce the modulus. Based on previous research [44,45], the industrial-grade sodium silicate was mixed with sodium hydroxide to adjust the sodium silicate modulus to 1.5. In this time, the alkali activators’ concentration is 37% [44,45]. The mortar liquid–solid ratio and the binder–sand ratio were designed at 0.8 and 1:3, accordingly. This study focuses on the effects of different GBFS dosing values on the mechanical properties and durability of the MK–GBFS geopolymer mortar. The flowchart of this study is displayed in Figure 3.

### 2.3. Methods

#### 2.3.1. Mass Change Rate

The mass changes of the mortar were tested according to GB/T749-2008 [45]. MK–GBFS geopolymer mortars were subjected to standard curing for 28 d, followed by immersion in the composite erosion solution. Then, their mass changes were measured at 30 d, 60 d, 90 d, and 120 d after immersion in a 5%${\mathrm{Na}}_{2}{\mathrm{SO}}_{4}$ + 5%NaCl solution. The rate of mass change was calculated according to Equation (1).

(1) $$\Delta m=\frac{m_{0}-m_{t}}{m_{t}}\times 100\%$$

where Δm is the mass of specimens with erosion, $m_{0}$ is the mass of specimens without erosion, and $m_{t}$ is the mass of specimens after erosion.

#### 2.3.2. Mechanical Properties

**Figure 4:**
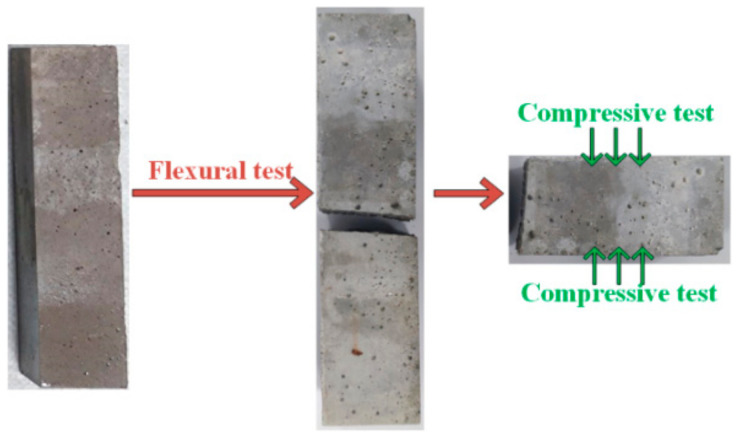
Mechanical property test methods.

The mechanical property measurement using the YAW–300C (Zhejiang, China) microcomputer-controlled compressive flexural testing machine is shown in Figure 4, according to GB/T17671–2021 [46]. The flexural strength of the mortar for all the specimens measuring 40 mm × 40 mm × 160 mm was analyzed. The compressive strength measurement was conducted using the fractured specimens in the flexural test, and the center of the specimen was ±0.5 mm from the press center. In particular, the compressive strength under semi-immersion was obtained from the average of the strengths of the wet and dry zones.

#### 2.3.3. XRD and SEM

The specimens’ microstructures were detected using the D8–Advance (Bremen, Germany, BRUKER) X-ray diffractometer (XRD) and observed utilizing the Sigma–300 (Jena, Germany, ZEISS) scanning electron microscope (SEM). After completing the mechanical strength test, all the samples were subjected to termination hydration treatment immediately for 3 d to suspend hydration. Subsequently, they were placed in an electric blast oven for drying. After that, they were ground. The specimens were crushed into 4–8 mm pieces for SEM analysis. The powder used for XRD was sieved with a 0.075 mm sieve. The XRD was operated at 30 kV, and the range was 5–90° with a speed of 5°/min.

## 3. Results

### 3.1. Apparent Phenomenon

**Figure 5:**
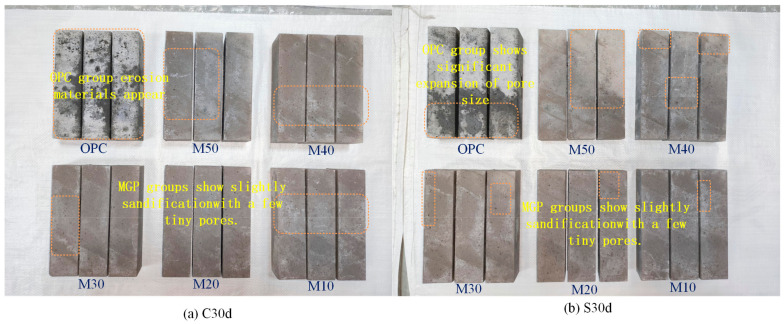

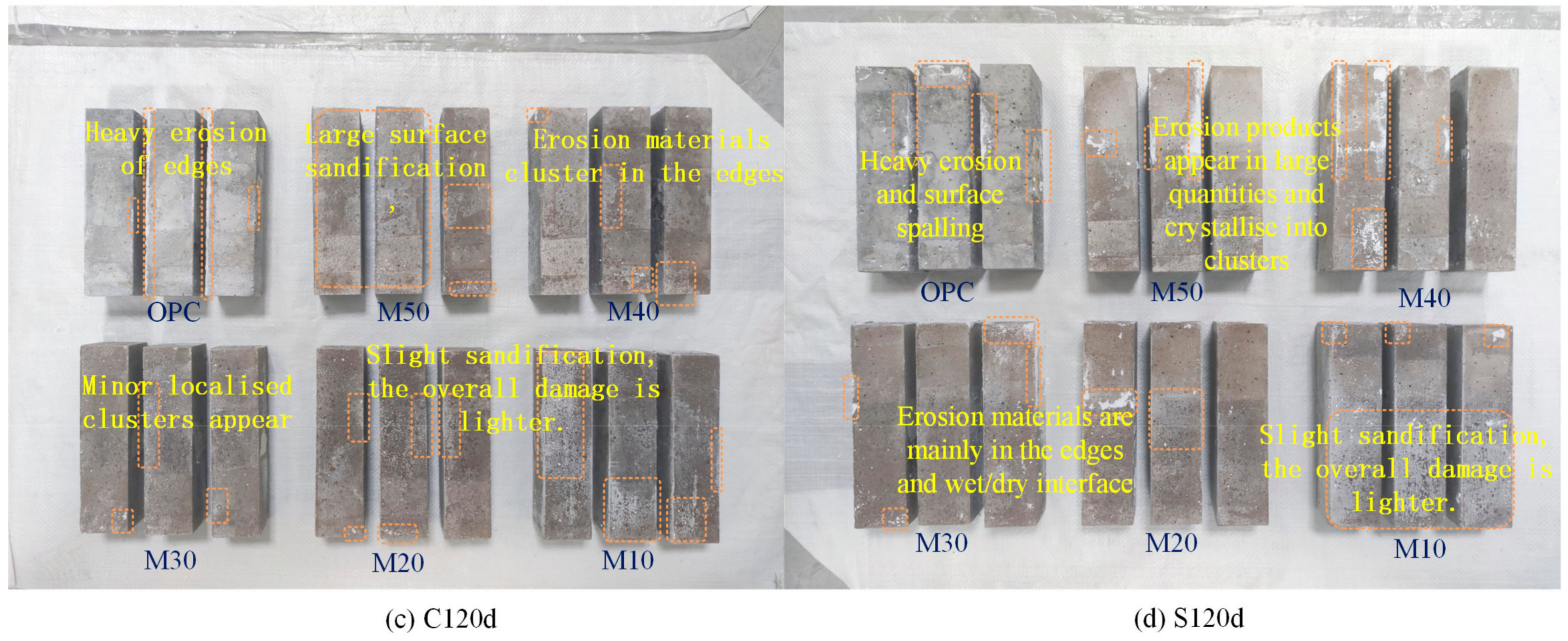
Apparent morphology of mortar specimens at 30 d and 120 d.

Figure 5 shows the apparent phenomenon of the specimens immersed in chloride–sulfate erosion for 30 d and 120 d. The specimens in the metakaolin geopolymer (MGP) groups show only slight sandification during the 30 d of erosion, while white erosion materials appear on the surface of the OPC group. The specimens immersed in chloride–sulfate erosion for 120 d are attached to different degrees of white erosive substances. Moreover, a small number of holes exist on their surfaces, but no significant cracks are detected. With the increase in GBFS content, a large amount of white erosion materials is observed. The semi-immersion specimens exhibit obvious immersion–evaporation interface zones due to the existence of the ‘wick effect’, leading to the formation of white erosion materials after the evaporation of water [47]. Under complete-immersion, these white eroded materials appear mostly near the edges and pores, while they are distributed at the immersion–evaporation interface in the form of localized agglomerates under semi-immersion.

### 3.2. Mass Change Rate

**Figure 6:**
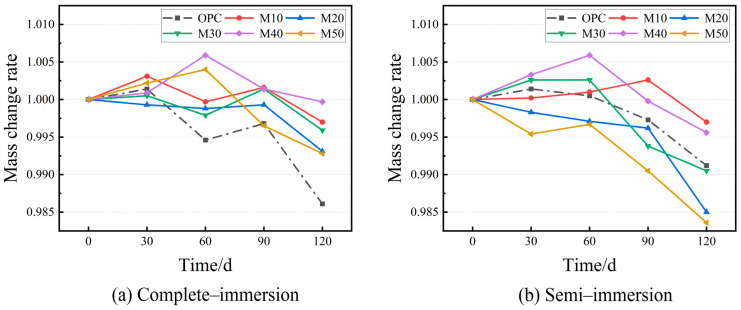
Mass change rate of the mortar specimens with age.

Figure 6 shows the results of the cement and geopolymer mortars that were immersed in chloride–sulfate erosion to evaluate the mass changes in each group over 120 d. Throughout the entire corrosion process, except for the M20 group, all the mortars’ masses exhibit a rising trend in the complete/semi-immersion, while their masses decrease in the late stage. In complete immersion, the mass changes in the MGP groups are all within 1%, which is significantly lower than that of the OPC group. It is concluded that the MGP group is less affected by the erosion of complete immersion in chloride–sulfate than the OPC group. Furthermore, the M20 and M50 groups have the highest mass change rate at 120 d in the semi-immersion, with the mass change rate of the specimens exceeding 1.5%, indicating that some of the MGP groups are more exposed to erosion in different immersion modes.

### 3.3. Mechanical Properties of the Specimens under Different Immersion Methods

#### 3.3.1. Mechanical Properties

**Figure 7:**
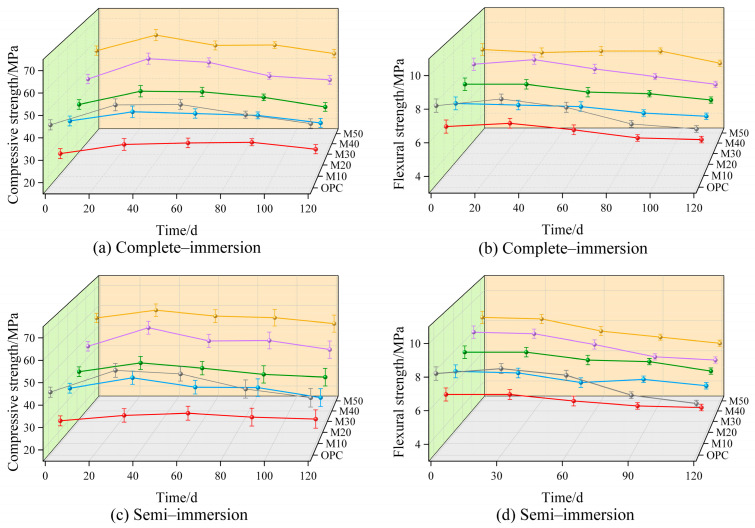
Strength of the mortar specimens with age.

The mechanical properties of the specimens under complete-immersion are depicted in Figure 7. Before corrosion, the compressive and flexural strength of M50 is as high as 56.0 MPa and 8.5 MPa, which are 12.6 MPa and 0.6 MPa higher than those of OPC and are 120% and 41% higher than those of M10, respectively. In addition, they show slight growth in chloride–sulfate erosion, whereas they show a decrease during the complete immersion erosion period. It is worth noting that M40’s compressive and flexural strengths are 4.5% and 24.1% higher than those of the OPC group after 120 d of erosion, respectively. As for M50, its compressive and flexural strengths are 4.6% and 16.9% higher than those of the OPC group specimens during the same erosion period.

#### 3.3.2. Corrosion Resistance Coefficient

**Figure 8:**
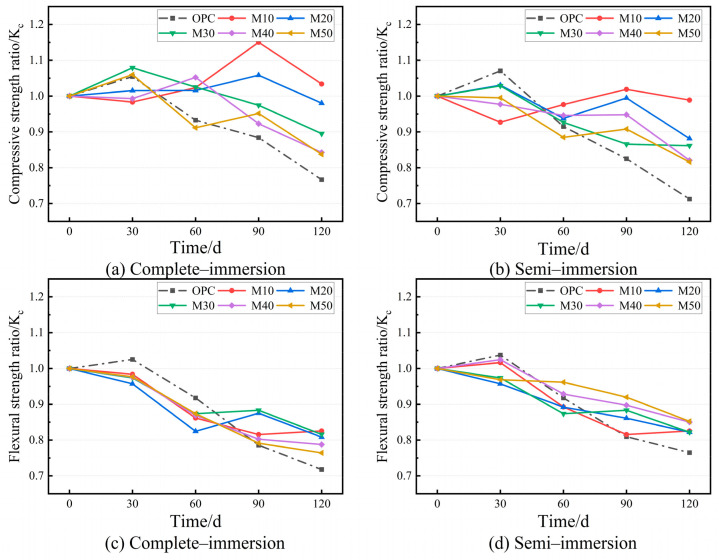
The corrosion resistance coefficient with age.

The strength ratio with different erosion ages in complete immersion is shown in Figure 8. The compressive strength ratio and flexural strength ratio of the OPC and most MGP groups in complete immersion in chloride–sulfate erosion at 120 d is reduced. It is noted that the compressive strength and flexural strength ratio of the MGP groups increase and are above 0.8, compared with the OPC group (0.77 and 0.76). Especially the M10 group with added low GBFS and the highest MK dosage show an increase in compressive strength at 120 d. This is ascribed to the formation of a three-dimensional mesh structure from the high Al and Si content of MK, and the low GBFS admixture lacks the Ca need for expansive erosion products [47,48,49].

After immersion for 120 d in both the complete-immersion and semi-immersion environments, the compressive strength varied similarly in each group, while the flexural strength changed significantly, as shown in Figure 7 and Figure 8. Furthermore, this difference is distinct as the GBFS dosage gradually rises, suggesting that the chloride–sulfate erosion in the solid–liquid interaction zone occurs more drastically compared with complete immersion [50]. For all the specimens, the destructive effects of chloride–sulfate erosion are stronger in the semi-immersion environment than in the complete-immersion environment. In addition, the increase in GBFS dosage compared with complete immersion has a significant effect on the flexural strength ratio in the semi-immersion.

#### 3.3.3. Mechanical Properties of the Immersion and Evaporation Zone under Semi-Immersion

**Figure 9:**
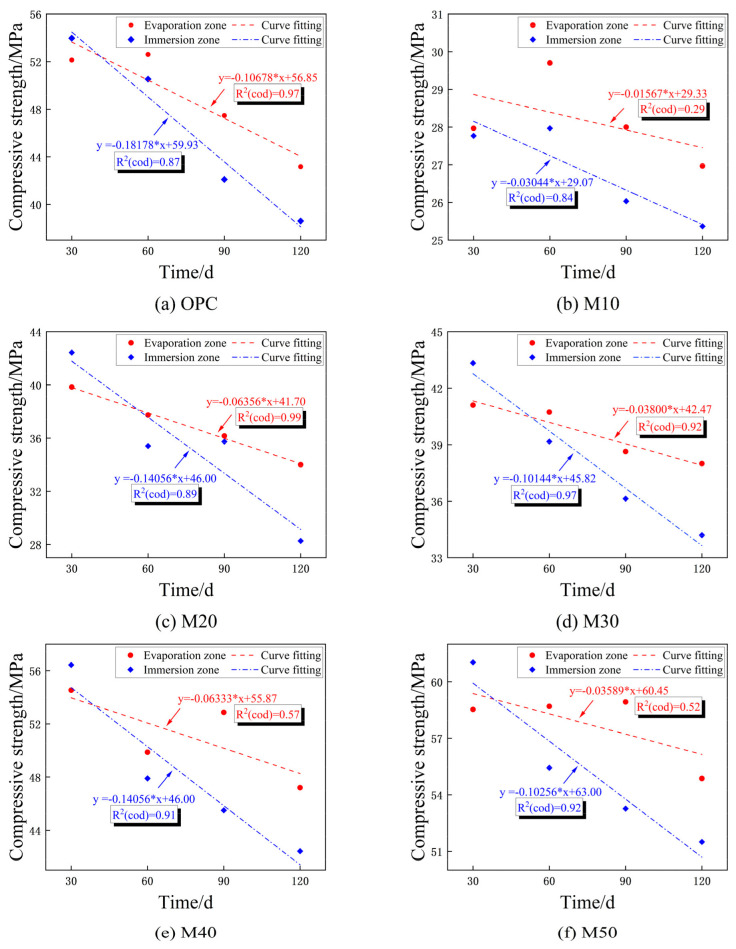
Compressive strength deterioration of the immersion and evaporation zones in semi-immersion.

Figure 9 show the compressive strength of the test groups’ zones (immersion and evaporation regions) using vertical semi-immersion. The compressive strength of the immersion and evaporation zones is linearly fitted to provide a more intuitive view of the deterioration pattern of the compressive strength. The deterioration of all specimens in the immersion zone is more serious; the slope ranges from −0.03 to −0.19, which is greater than the slope of the fitted curve for the evaporation zone (−0.01 to −0.11). Compared with the immersion zone, matrices lose less compressive strength and erode slightly in the evaporation zone. In addition, the slopes of the fitted curves for both the immersion and evaporation zones of the MGP groups are much lower than those of the OPC group and increase followed by a decrease in the presence of GBFS.

### 3.4. XRD Analysis

**Figure 10:**
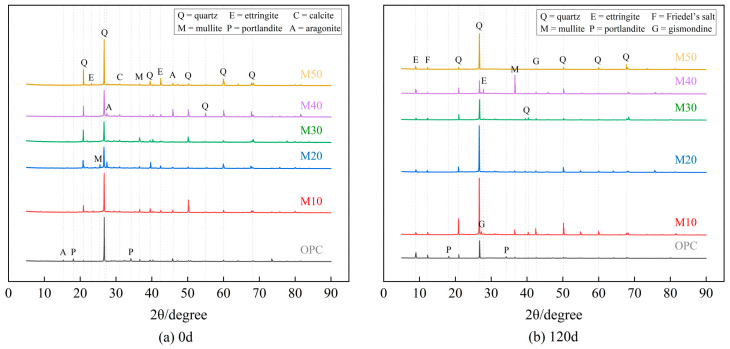
XRD images at different times.

As shown in Figure 10, gypsum is not found in both the OPC or MGP mortar specimens at 0 d and 120 d of erosion, indicating that gypsum only appears as an intermediate product in the middle stage of erosion because its structure is simpler than AFt. However, AFt with a more stable structure is formed because of the lower thermodynamic free energy in the later stages of erosion, which is the main factor causing expansive erosion damage in the later stages. Moreover, the MGP groups with high GBFS dosage have a higher Ca content, and the microstructures of the products are different after 120 d of erosion. Therefore, their erosion resistance in chloride–sulfate erosion is still improved compared with that of the OPC group [50,51].

### 3.5. SEM Analysis

**Figure 11:**
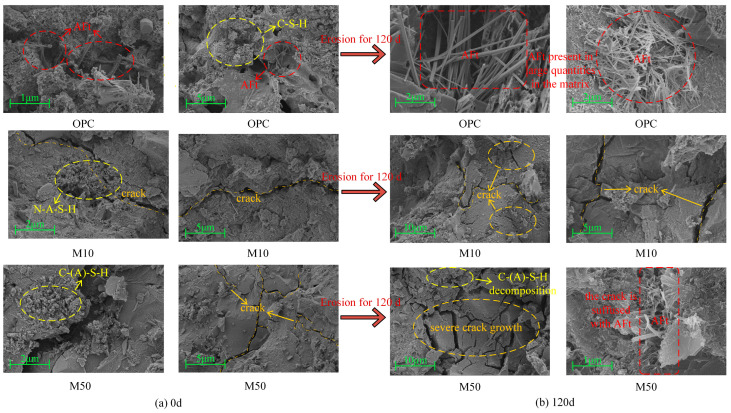
SEM images at different times.

Figure 11 presents the SEM images of the mortar specimens from the OPC and MGP groups after immersion for 0 d and 120 d in the chloride–sulfate solution. Before erosion, the cracks are narrower in width and are surrounded by a large amount of dense hydration products (C–(A)–S–H, N–A–S–H) bridging the cracks. A limited amount of AFt generation is found in the OPC group. With the development of erosion, the OPC group and the M50 group generate a large amount of acicular AFt, causing expansive erosion damage. The erosion product AFt expands and accumulates inside the mortar, resulting in expansion stress exceeding the bearing limit of the matrix, and then new microcracks occur. Subsequently, the mortar substrate sustains more serious damage when erosion ions enter the structure through the cracks [52,53]. In addition, both the OPC and MGP groups show rapid crack growth after 120 d of erosion, and their internal structure deteriorates seriously, demonstrating the decomposition of hydration products due to the continuous penetration of erosion ions into the matrix.

## 4. Discussion

**Figure 12:**
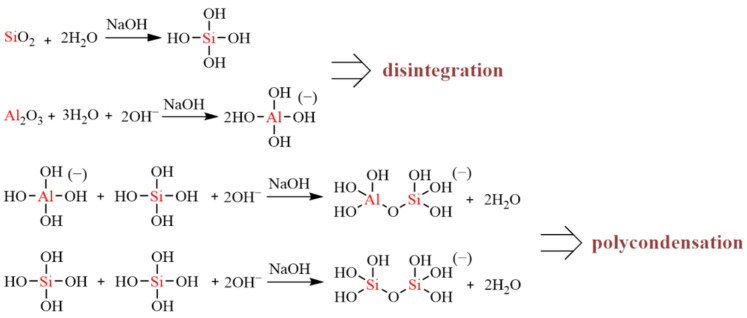
Geopolymer disintegration and polycondensation.

The change in mechanical properties of geopolymer mortar can be divided into two stages according to the disintegration and polycondensation reaction of geopolymer, and the reaction process is illustrated in Figure 12. The disintegration process proceeds when the raw materials release Al and Si to form the free state of monomers. The polymerization of these monomers in the alkaline environment constituted by the sodium silicate solution occurs rapidly to form Al–O–Si and Si–O–Si bonds, leading to the formation of a three-dimensional mesh structure with strength [54]. In addition, the Ca/Si ratio and CaO increase inside the matrix with the increase in the GBFS dosage. The calcium content of the MGP groups (M40 and M50) is closer to OPC. However, compared with OPC, a large amount of MK can provide Al, which causes the reactions of the matrix and the formation of the C–(A)–S–H gel and N–A–S–H gels. The filling effect formed inside the matrix makes it denser and further enhances its strength [55,56,57].

**Figure 13:**
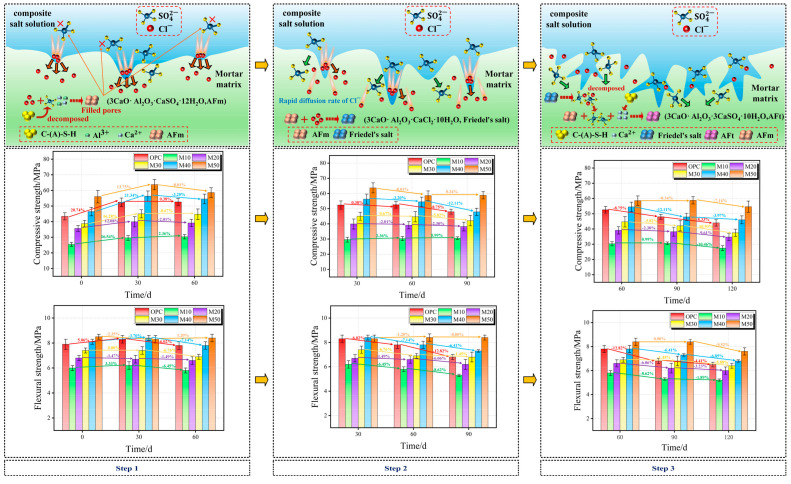
Schematic diagram of the corrosion model.

The mechanical strength of specimens under chloride–sulfate erosion initially increases and subsequently decreases, owing to continuous hydration and chloride–sulfate erosion. As shown in Figure 13, the mortar specimens are formed into Ca_4_Al_2_(SO_4_)(OH)_12_·6H_2_O (AFm) and fill up the pore space in the cementitious material because the diffusion rate of ${\mathrm{Cl}}^{-}$ is greater than ${\mathrm{SO}}_{4}^{2-}$, suggesting that chlorides may inhibit sulfate erosion in the early stage. Furthermore, the coexistence of ${\mathrm{Cl}}^{-}$ and ${\mathrm{SO}}_{4}^{2-}$ compete with AFm, and then ${\mathrm{Cl}}^{-}$ binds AFm rapidly, leading to the production of Friedel’s salt. Therefore, the pore space further is refined in the matrix, inhibiting ${\mathrm{SO}}_{4}^{2-}$ erosion [58]. With increasing erosion time, AFm and Friedel’s salt are converted into more stable AFt, in which Friedel’s salt combines with ${\mathrm{SO}}_{4}^{2-}$ to produce more stable AFt and release solidified ${\mathrm{Cl}}^{-}$ [37,59,60]. Moreover, the solubility of the erosion product AFt in the chloride solution is three times that in water, and the fast diffusion of ${\mathrm{Cl}}^{-}$ alleviates the generation of the expansive erosion product AFt to reduce the expansion of the microcracks in the early stage of the specimen during the pre-erosion period, along with Friedel’s salt inside the matrix. In the early stage of erosion, ${\mathrm{Cl}}^{-}$ inhibits the erosion of ${\mathrm{SO}}_{4}^{2-}$. The related reaction process is expressed as follows in Equations (2)–(4):

(2) $$\begin{array}{l} 3\mathrm{CaO}•{\mathrm{Al}}_{2}O_{3}•{\mathrm{CaSO}}_{4}•{12H}_{2}{O+2\mathrm{SO}}_{4}^{2-}+2{\mathrm{Ca}}^{2+}+20H_{2}O\\ \;\to 3\mathrm{CaO}•{\mathrm{Al}}_{2}O_{3}•{\mathrm{CaSO}}_{4}•32H_{2}O \end{array}$$

(3) $$\begin{array}{l} 3\mathrm{CaO}•{\mathrm{Al}}_{2}O_{3}•{\mathrm{CaSO}}_{4}•{12H}_{2}{O+2\mathrm{Cl}}^{-}\\ \;\to 3\mathrm{CaO}•{\mathrm{Al}}_{2}O_{3}•{\mathrm{CaCl}}_{2}•{10H}_{2}{O+\mathrm{SO}}_{4}^{2-}{+2H}_{2}O \end{array}$$

(4) $$\begin{array}{l} 3\mathrm{CaO}•{\mathrm{Al}}_{2}O_{3}•{\mathrm{CaSO}}_{4}•H_{2}{O+2\mathrm{SO}}_{4}^{2-}{+2\mathrm{Ca}}^{2+}{+20H}_{2}O\\ \;\to 3\mathrm{CaO}•{\mathrm{Al}}_{2}O_{3}•3{\mathrm{CaSO}}_{4}•32H_{2}O \end{array}$$

As the age of erosion progresses, the matrix begins to develop microcracks, which further accelerates the diffusion of ${\mathrm{Cl}}^{-}$ and ${\mathrm{SO}}_{4}^{2-}$. The expansive erosion products damage the pore structure, and the eroded ions react with the gel material (N–A–S–H, C–A–S–H) in the mortar, which causes the structure to swell and crack, resulting in a significant loss of strength [61,62]. In addition, as the GBFS dosage increases, the rate of decrease in strength after 120 d of erosion gradually increases. In the low-GBFS dosage MGP groups, there are more MK particles containing a large amount of Al and Si, leading to the formation of a zeolite-like three-dimensional mesh structure for the adsorption of ${\mathrm{Ca}}^{2+}$. Furthermore, ${\mathrm{Ca}}^{2+}$ is also adsorbed in the N–A–S–H gel through the charge balance effect, reducing the generation of the C–A–S–H gel. The fast diffusion ${\mathrm{Cl}}^{-}$ are the first to generate AFm and Friedel’s salt in the vicinity of the N–A–S–H gel, which makes it difficult for ${\mathrm{SO}}_{4}^{2-}$ to generate AFt and other swelling erosion materials, slowing down the erosion rate [63,64].

The mechanical properties of the mortar in the MGP group are positively correlated with the GBFS dosage, and the C–(A)–S–H gel generated from its dissolution in the organism is denser than the N–A–S–H gel since GGBS is rich in Ca, refining the pore structure. Therefore, the mechanical properties of the mortar in the MGP groups are enhanced with the increase in the GBFS dosage, similar to the findings presented by Džunuzović [63] et al. However, the ${\mathrm{Ca}}^{2+}$ content rises with the increase in the GBFS dosage, promoting the generation of C–A–S–H gel. In the presence of erosive material, AFt is further generated with expansion, and the generated material accumulates, which allows the expansion pressure to surpass a threshold, inducing swells and cracks in the hardened slurry. Thus, the reduction in compressive strength is obvious, further indicating that the increase in the GBFS dosage in the MGP groups results in accelerated erosion [65,66]. As the GBFS dosage increases and the matrix densifies, the supersaturation concentration required to produce salt crystals rises concomitantly, as well as the crystallization pressure generated, increasing the potential for erosion. However, while simultaneously benefiting from the early densification of the matrix, it still exhibits a high level of mechanical strength following erosion.

**Figure 14:**
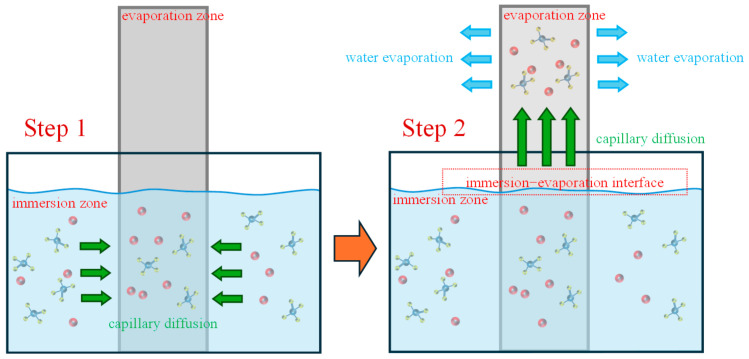
Semi-immersion ion transfer.

Similar to complete immersion, chlorides inhibit and then promote sulfate erosion under the effect of semi-immersion with chloride–sulfate erosion. In addition, as shown in Figure 14, the erosion medium in the immersion zone is subject to capillary migration as a result of the ion concentration difference and the ‘wick effect’. This migration occurs towards the immersion-evaporation interface, where it accelerates the ion migration and precipitates crystals in the evaporation zone as a consequence of the evaporation of water. Given that the immersion-evaporation interface is situated at the junction between the saturated and unsaturated zones, it gives rise to a stacking effect in the region during the process of erosion and ion transport. Consequently, the flexural test is loaded at the immersion-evaporation interface, which results in a more pronounced decline in flexural strength relative to compressive strength [67,68].

In this paper, a comparative analysis is conducted between the erosion of each group with 10–50% slag doping and existing research findings. This study emphasizes the analysis of the erosion mechanisms under chloride–sulfate erosion, employing a combination of quantitative and qualitative research methods to investigate these mechanisms. The results show that the M40 and M50 groups have better mechanical properties and erosion resistance compared with the OPC group, establishing a certain foundation for the engineering application of geopolymer materials in highly corrosive environments (chloride–sulfate erosion), such as coastal areas, acid rain lakes, and other highly erosive areas.

## 5. Conclusions

In this study, the erosion resistance of the OPC group and MGP groups under complete/semi-immersion in a 5%${\mathrm{Na}}_{2}{\mathrm{SO}}_{4}$ + 5%NaCl solution environment is studied. According to the test and analysis results, the conclusions are as follows:

(1) In the chloride–sulfate erosion environment, the M50 group and the OPC group erode significantly, with surface spalling and white erosion products. The degree of erosion is markedly diminished at a low GBFS dosage. In addition, the OPC group exhibited the most pronounced mass loss under complete immersion, in comparison to the MGP groups, which demonstrated heightened sensitivity and experienced significant mass loss under semi-immersion.
(2) The initial compressive and flexural strengths of the M50 group can reach 73.58 and 10 MPa, respectively, which represent a 62.14% and 62.14% increase in comparison with the OPC group. After complete-immersion corrosion, the residual compressive and flexural strengths of the M50 group are 55.71 MPa higher than those of the OPC group. Similarly, the residual compressive and flexural strengths of the M50 group are observed to be higher than those of the OPC group under semi-immersion. Compared with the OPC group, the MGP group with its three-dimensional mesh structure and low-calcium system demonstrates an effective capacity to impede the erosion of chloride–sulfate.
(3) Compared with complete-immersion, semi-immersion demonstrated a more pronounced behavioral response, characterized by extensive damage to the matrix at the immersion-evaporation interface zone. This was accompanied by a notable reduction in flexural strength, attributed to the ionic concentration disparity and the ‘wick effect’. By calculating the compressive strength of both the immersion and evaporation zones of the specimens, it was determined that the slope of strength loss in the immersion zone is significantly greater than that in the evaporation zone. Thus, it can be concluded that the erosion in the immersion zone is more severe.
(4) In the initial phase of chloride–sulfate erosion, the rapid diffusion of ${\mathrm{Cl}}^{-}$ with ${\mathrm{Ca}}^{2+}$ generated AFm and Friedel’s salt, impeding the erosion of ${\mathrm{SO}}_{4}^{2-}$. In the late stage of erosion, the expansion of microcracks leads to the generation of more stable AFt and the release of ${\mathrm{Cl}}^{-}$. The formation of a binding effect between the two erosion ions resulted in more severe erosion. The MGP group exhibited superior erosion resistance in comparison with the OPC group. However, the elevated GBFS dosage increased the risk of erosion while simultaneously enhancing the mechanical strength of the matrix. Under different conditions, the initial strength of the M40 and M50 groups was higher than that of the OPC group. Furthermore, their erosion resistances increased by 6–10% and 4–10% after 120 d of erosion, respectively. Therefore, the M40 and M50 groups are more suitable for practical engineering applications.
(5) This paper emphasizes the interaction between chloride and sulfate. However, the erosion rate and the depth of erosion ions under semi-immersion are not yet known, especially in the immersion-evaporation interaction zone. Further qualitative research is essential. In summary, the mechanisms of erosion resistance under complete/semi-immersion is an important future research direction, utilizing MK–GBFS-based polymers to achieve low carbon, high strength, and great durability.

## Figures and Tables

**Table 1:** The main chemical composition (wt.%).

Material	CaO	SiO_2_	Al_2_O_3_	Fe_2_O_3_	MgO	SO_3_	K_2_O
MK	0.65	51.59	40.05	2.30	2.17	0.41	1.57
GGBS	43.15	29.20	12.59	1.44	8.09	2.00	0.45
OPC	56.78	25.52	7.51	2.89	2.43	1.33	0.67

**Table 2:** Mix proportions of MK–GBFS-based geopolymers.

Sample	GGBS/%	Material/g			
		MK	GGBS	OPC	Activator
OPC	0	0	0	100	0
M10	10	405	45	0	10
M20	20	360	90	0	20
M30	30	315	135	0	30
M40	40	270	180	0	40

## Data Availability

The original contributions presented in the study are included in this article. Further inquiries can be directed to the corresponding author.

## Abbreviations

MK	metakaolin
GBFS	granulated blast furnace slag
OPC	Ordinary Portland Cement
MGP	metakaolin geopolymer
$K_{b}$	alkalinity coefficient
SEM	scanning electron microscope
XRD	X-ray diffractometer
AFt	Ettringite
AFm	Ca_4_Al_2_(SO_4_)(OH)_12_·6H_2_O
Δm	the mass of specimens without erosion
$m_{0}$	the mass of a specimen without erosion
$m_{t}$	the mass of a specimen after erosion
$K_{c}$	strength ratio
